# Is single-port video-assisted thoracic surgery for mediastinal cystectomy feasible?

**DOI:** 10.1186/s13019-019-0843-9

**Published:** 2019-01-22

**Authors:** Nanqing Jiang, Yiming Lu, Jun Wang

**Affiliations:** Department of Cardiothoracic Surgery, The First People’s Hospital of Changzhou, The Third Affiliated Hospital of Soochow University, 185 Juqian Road, Changzhou, 213003 Jiangsu China

**Keywords:** Mediastinal cystectomy, VATS, Single-port

## Abstract

**Background:**

Video-assisted thoracic surgery (VATS) for mediastinal cysts has been used with increasing frequency. Both single-port VATS and three-port VATS procedures are used for mediastinal cystectomy. Few studies have been published to compare three-port VATS and single-port VATS procedures in mediastinal cystectomy.

**Methods:**

Forty-five patients with mediastinal cysts who underwent single-port procedures (*n* = 23) or three-port procedures (*n* = 22) in our department from January 2016 to July 2018 were retrospectively analysed. The perioperative conditions and pathological findings were analysed.

**Results:**

The single-port group showed shorter operation times [45 (35–60) vs 55 (45–80) min, *p* = 0.013], less retention time of the thoracic drainage tube [27(24–48) vs 48(48–70) *p* < 0.001)], shorter postoperative hospital stays [5(4–6) vs 7(5–7), *p* = 0.011] and less costs [2.0)1.2–2.5) vs 2.5(1.9–3.5), *p* = 0.032] than those of the three-port group. No difference was found in case conversions to open procedures (*p* > 0.99) or second operations (*p* > 0.99). Logistic regression analysis showed that the surgical method (*p* = 0.426) and surgeon experience (*p* = 0.719) were not independent prognostic factors for the success of surgery.

**Conclusions:**

The single-port VATS procedure was not inferior to the three-port VATS procedure for mediastinal cystectomy. The single-port VATS procedure is a feasible choice for mediastinal cystectomy.

## Introduction

Cystic lesions account for 20–32% of all mediastinal lesions [1]. Most surgeons recommend surgical resection for mediastinal cysts [2]. Complete surgical excision is thought to be the standard therapy for mediastinal cysts [2]. The accuracy rate of the CT examination for mediastinal cysts is not 100% [3]. Some patients with mediastinal cysts have symptoms. The purpose of surgery is to make a definitive diagnosis and to improve symptoms in symptomatic patients.

Open surgery for mediastinal cysts has previously been widely used. With the development of minimally invasion surgery, video-assisted thoracic surgery (VATS) resection for mediastinal cysts has been used with increasing frequency. It has been reported that VATS resection for mediastinal cyst is feasible and safe [4, 5]. The three-port VATS procedure is the most frequently used approach for mediastinal cystectomies. Single-port VATS was first reported by Rocco et al. in 2004 [6]. With the development of the surgical technique and instruments, increasing numbers of single-port VATS procedures have been performed. Few studies have been published to compare the results of three-port VATS versus single-port VATS procedures in mediastinal cystectomy. Both three-port VATS procedures for mediastinal cysts and single-port VATS for mediastinal cystectomy have been performed in our department for a long time. This study aimed to compare the three-port VATS procedure and single-port VATS procedure for mediastinal cystectomy in terms of perioperative complications. We pose a hypothesis that the single-port VATS procedure might have advantages over the three-port VATS procedure.

## Patients and methods

From January 2016 to July 2018, patients with mediastinal cysts confirmed by a postoperative pathological examination, who underwent a three-port VATS or single-port VATS procedure in Changzhou First People’s Hospital, Soochow University, China, were enrolled in this retrospective study. The operations were all performed by skilled surgeons with working experience of more than 5 years. The choice of surgical procedure depended on the training experience of the particular surgeon. Some surgeons preferred to perform the single-port VATS method, while others preferred the three-port VATS procedure. Patients were excluded if they suffered from malignant diseases or other diseases requiring surgery.

All patients underwent history-taking and physical examinations before surgery. All patients underwent pulmonary function testing, electrocardiographic examinations, blood tests, and CT scanning of the thorax. This study was approved by the Clinical Research Ethics Committee of our hospital.

### Surgical procedure

**Fig. 1:**
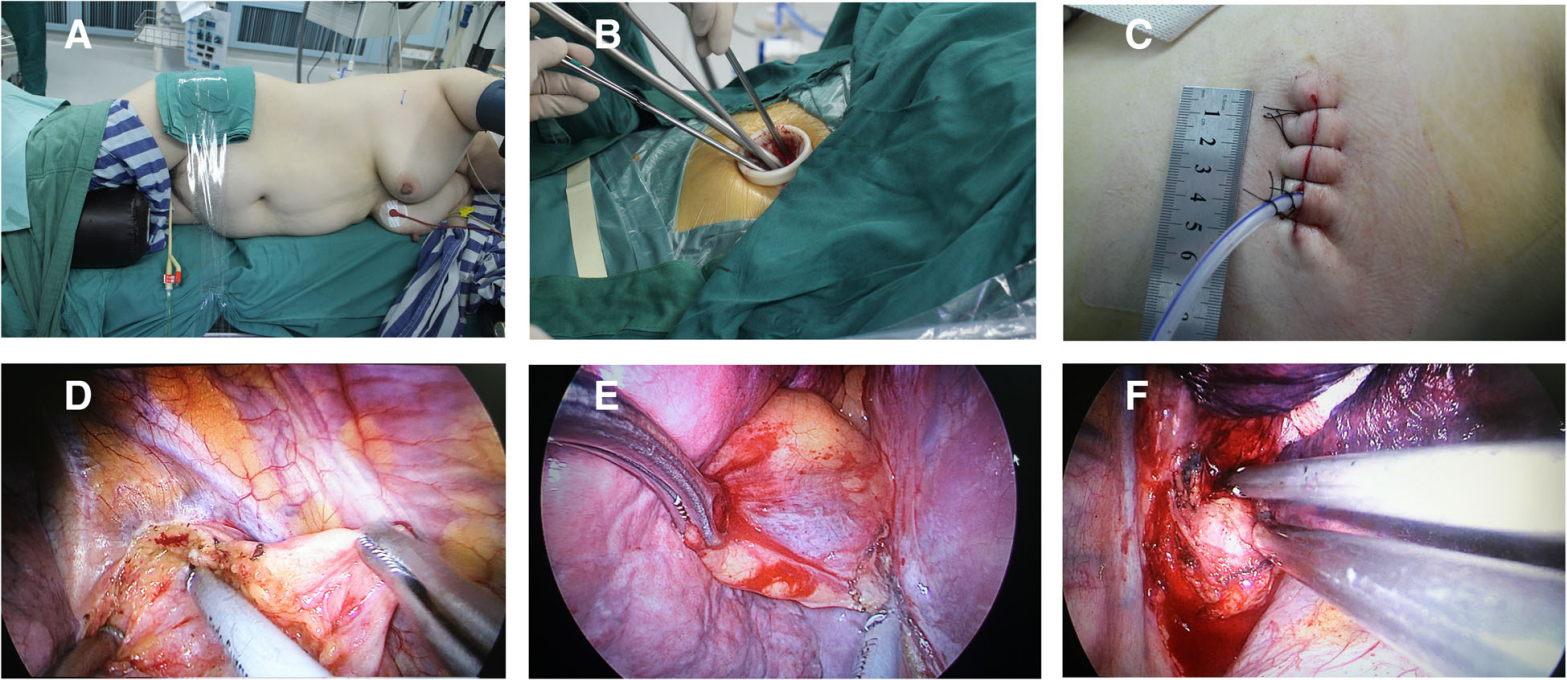
**a** Patient’s position; (**b**) and (**c**) the operative incision and incision length. **d**, (**e**) and (**f**) Method for addressing the lesions. **d** Method for addressing a thymic cyst; (**e**) method for addressing a pericardial cyst; and (**f**) method for how to address bronchogenic cysts

The single-port VATS procedure was performed as described below. Patients were placed in the lateral position (Fig. 1a) under general anaesthesia with a tracheal double-catheter insertion. The left lateral position was the most often-used position for these patients. The right lateral position was used if the mediastinal cyst was in the left mediastinum. A 4–5 cm incision was made in the fifth intercostal space in the anterior axillary line on the chest for the surgical approach (Fig. 1b,c). An aspirator and ultrasonic scalpels were used to dissect the cyst along the cyst edge. A double-joint sponge forceps was used to help expose the surgical field if necessary (Fig. 1d, e, f). A negative pressure absorbing ball (Pupunch, BDA-YS 0200, Baiduo Medical Corporation, Shandong, PRC) was used for chest drainage after the operation (Fig. 1c).

The three-port VATS procedure is described below. Patients were placed in the lateral position. The left or right lateral position depended on the location of the lesion. A 1.5 cm observation hole was made in the seventh/eighth intercostal space in the midaxillary line. Two 1.5 cm incisions were made in the anterior axillary line and linea scapularis as the two operation holes. An aspirator and ultrasonic scalpels were used to dissect the cyst as in the single port procedure. A 28F thoracic tube (Zhanjiang star enterprise, Guangdong, PRC) was used for chest drainage after surgery.

### Cyst diameter and pathological examinations

The cyst diameter was measured by a CT scan before surgery. The surgical specimens were immersed in 4% formalin for 24 h and were stained with haematoxylin and eosin (HE). The cyst types were judged by skilled pathologists.

### Statistical analysis

Data are presented as the median value (quartile 1–quartile 3). A commercially available statistical software package SPSS 22.0 (SPSS, Inc. IL, USA) was used. Categorical variables were compared using the chi-square test. Numerical variables were compared using the t test. Statistical significance was defined as *P* < 0.05.

## Results

### Patient characteristics and laboratory findings

Forty-five mediastinal cyst patients who underwent the single-port VATS procedure (*n* = 22) and three-port VATS procedure (*n* = 23) in our department were included in this retrospective study. Clinical data are listed in Table 1. No statistically significant differences were found between the groups in terms of gender, age, body mass index (BMI), preoperative symptoms, forced expiratory volume in one second (FEV1.0), forced expiratory volume in one second/forced vital capacity (FEV1.0/FVC), PaO_2_, serum creatinine, blood glucose or cyst location before surgery.

**Table 1:** Patient characteristics and laboratory findings

	Single-port (*n* = 23)	Three-port (*n* = 22)	*P*
Gender Male:Female	11:12	12:10	0.652
Median age / years	54.0 (46.5–61.5)	54.0 (43.0–60.0)	0.686
BMI	24.2 (20.7–28.2)	24.2 (23.6–25.5)	0.914
Preoperative symptom (case)			
Yes	4	7	0.260
No	19	15	
FEV1.0/FVC / %	83.7 (76.7–87.9)	88.0 (83.0–90.8)	0.191
FEV1.0 /L	2.62 (2.38–2.97)	2.74 (2.38–3.29)	0.737
PaO_2_ /mmHg	82.0 (79.0–99.0)	89.0 (77.0–96.5)	0.378
Serum creatinine /μmol ·L^−1^	66.0 (56.0–78.0)	64.5 (56.0–87.3)	0.333
Blood glucose / mmol ·L^−1^	5.35 (4.97–6.13)	5.17 (4.49–5.70)	0.191
Lesion location			
anterior: middle: posterior	16:1:6	15:2:5	0.805

*BMI* body mass index*, FEV1.0* Forced expiratory volume in 1 s, *FVC* Forced expiratory volume, *EF* Ejection Fraction

### Perioperative conditions of the patients

The perioperative conditions of these patients are presented in Table 2. No statistically significant differences were found between the groups in terms of haemorrhage during surgery, drainage over 24 h, case conversions to open procedures, second operations, blood vessel injuries, pleural adhesions, duration of intensive care unit stays, number of postoperative thoracenteses, pain scores before extubation, pain scores after extubation or cases of painkiller administration. The pain score was estimated by facial expression of pain. The single-port group had shorter operation times [45 (35–60) vs 55 (45–80) min, *p* = 0.013], less retention time of the thoracic drainage tube [27(24–48) vs 48(48–70)], *p* < 0.001),shorter postoperative hospital stays [5(4–6) vs 7(5–7), *p* = 0.011] and less cost [2.0(1.2–2.5) vs 2.5(1.9–3.5), *p* = 0.032] than those of the three-port group. Surgeon experience was assessed by years of working. No difference was found in surgeon experience between the groups (*p* = 0.155).

**Table 2:** Perioperative conditions of the patients

	Single-port (*n* = 23)	Three-portal (*n* = 22)	*P*
Operation times / min	45 (35–60)	55 (45–80)	0.013
Haemorrhage during surgery/ mL	36 (10–72)	50 (20–100)	0.819
Drainage over 24 h / mL	100 (37.5–225)	100 (100–200)	0.893
Retain time of Thoracic drainage tube /h	27 (24–48)	48 (48–70)	< 0.001
Postoperative hospital stays / day	5 (4–6)	7 (5–7)	0.011
Case conversions to open	0	1	> 0.99
Second operations	1	0	> 0.99
Blood vessel injuries	0	1	> 0.99
Pleural adhesions	2	3	0.665
Duration of intensive care unit stays/h	30 (24–48)	36 (36–48)	0.117
Number of postoperative thoracenteses	2	0	0.489
Pain scores before extubation	2 (0–3.75)	2 (2–6)	0.180
Pain scores after extubation	0 (0–2)	0 (0–2)	0.488
Cases of painkiller	13	13	0.862
Surgeon experience (5–10 years: > 10 years)	11:12	6:16	0.155
Cost (ten thousand yuan/RMB)	2.0 (1.2–2.5)	2.5 (1.9–3.5)	0.032

### Pathological findings after surgery

The postoperative pathological findings are listed in Table 3. No significant differences were found in terms of the pathologic types of the cysts (*p* = 0.195) or maximum diameters of the cysts (*p* = 0.447). The relationship between the cyst location and pathological pattern was also analysed. Most bronchogenic cysts were in the posterior mediastinum, and most thymic and pericardial cysts were in the anterior mediastinum (*p* < 0.001) (Table 4).

**Table 3:** Pathological findings after operation

	Single-port (*n* = 23)	Three-portal (*n* = 22)	*P*
Pathologic types of cyst			0.195
Thymic cyst	7	5	
bronchogenic cyst	7	9	
Pericardial cyst	9	5	
Simple cyst	0	3	
Maximum diameter of cysts /cm	3.9 (2.8–4.1)	5.0 (3.0–6.0)	0.447

**Table 4:** Relationship between cyst location and pathological pattern

	Anterior	Middle	Posterior	*P*
Thymic cyst	12	0	0	< 0.001
bronchogenic cyst	5	1	10	
Pericardial cyst	13	1	0	
Simple cyst	1	1	1	

### Factors influencing the success of surgery

We measured procedure success as complete resection of cysts and no conversion to open surgery or performance of a second operation. Logistic regression analysis was performed to identify the factors that influenced the success of surgery by adjusting the variables chosen in advance and based on clinical relevance, including the operation time, cyst location, diameter of the cysts, surgeon experience and surgical procedure (Table 5). Surgeon experience was assessed by years of working. Logistic regression analysis showed that the operation time (*p* = 0.690), cyst location (*p* = 0.958), diameter of cysts (*p* = 0.488), surgical method (*p* = 0.426) and surgeon experience (*p* = 0.719) were not prognostic factors in these patients.

**Table 5:** Results of logistic regression analysis for factors influencing the success of surgery

	B	P	OR	95% CI for OR	
				upper	lower
intercept	−3.280	0.141			
Operation time	−0.011	0.690	0.989	0.937	1.044
Cyst location	−0.071	0.958	0.932	0.066	13.201
Diameter	0.186	0.488	1.205	0.712	2.038
Surgical approach	1.131	0.426	3.098	0.192	50.045
Surgeon experience	−0.508	0.719	0.602	0.038	9.570

## Discussion

Only one 4–5 cm incision was needed in the single-port procedures, while three 1–2 cm incisions were needed in three-port procedures. Making the extra incisions was time-consuming. The three small incisions also required more time to achieve haemostasis and closing. A single 4–5 cm incision more easily achieved haemostasis and closing than did the three small incisions. Our results showed that shorter operation times were required in the single-port group than in the three-port group.

The thoracic drain was removed when drainage was no more than 150 ml over 24 h. It has been reported that small and large chest tubes are both effective for pleural effusions [7],although the small-size chest tube might suffer from blockage [8]. The tube of Pupunch could be placed in the surgical field, while the normal thoracic tube could not be placed in the surgical field. The negative pressure of the Pupunch tube could also promote surgical field closure. The blocking of the drainage tube led to pleural effusion. A chest film was routinely performed for patients before discharge. If a pleural effusion was found on the chest X-ray, B-mode ultrasonographic scanning was performed. Thoracenteses was performed for patients with medium or more than medium pleural effusion diagnosed on ultrasound. Two patients received postoperative thoracenteses in single-port group. One was asymptomatic, the other was suffered from low-grade fever. Our results showed that more patients required postoperative thoracenteses in the single-port group than in the three-port VATS group (2/23 vs 0/22, *p* = 0.489), with no significantly difference between the groups.

Our results also showed lower costs in the single-port VATS group than in the three-port VATS group. Only an incision protector (model QXB-A 0607, Kangxin Medical Corporation, Changzhou, PRC) was used in the single-port group. Two small-sized incision protectors (model HK-50/40–25/25(D), Kadi Medical Corporation, Beijing, PRC) and a 12 mm universal trocar stability sleeve (Johnson and Johnson, USA) were needed in the three-port group. The single-port group also had shorter postoperative hospital stays as well as shorter durations of intensive care unit stays. For these reasons, the single-port group was more economical than the three-port group.

It has been reported that there are no differences in pain scores between large and small drain groups [8]. Another study showed that less pain occurred with the use of small-sized thoracic tubes [9]. Only one intercostal nerve might be injured during the single-port VATS procedure, while three intercostal nerves might be injured in three-port procedure. The sense of pain varies among people, and the measurement of pain is not precise. No difference was found in pain scores before or after extubation in this study.

One case in the three-port group was converted to open surgery because of injury to the brachiocephalic vein. No conversion occurred in the single port-group, which means the single-port procedure was also safe for patients with mediastinal cysts. The total incidence of conversion was 2.2% (1/45). Haemostasis by direct compression was achieved as soon as possible in this case. The patient was then changed to the horizontal position and a median sternotomy was performed for further haemostasis.

One case in the single -port group underwent a second operation because of postoperative bleeding. The three–port procedure was adopted for this second operation. The reason for postoperative bleeding was incision bleeding. Incision bleeding is more difficult to observe during the single–port procedure than during the three–port procedure, which may be a major defect of the single–port procedure.

It has been reported that bronchogenic cysts were the most common type of primary cyst in the mediastinum [4]. Thymic cysts have been reported to be another type of common mediastinal cyst [10–12]. Previous studies also showed that pericardial cysts accounted for 18–20% of all mediastinal cysts [12, 13]. Our findings agreed with the results reported in the literature. Simple cysts are a rare type of mediastinum cysts. There have been very few reports of the incidence of simple mediastinal cysts. The incidence of simple cysts in our study was 6.6% (3/45). Most bronchogenic mediastinal cysts are located in the middle or posterior areas of the mediastinum [14]. Our results also showed that most of the bronchogenic cysts were located in the posterior mediastinum. Thymic cystsare located in the developmental line of the thymus and in the anterior mediastinum [3]. All thymic cysts were located in the anterior mediastinum in our study. Most pericardial cysts were located along the cardiac border and in the anterior mediastinum [15]. Our results also showed that most pericardial cysts were located in the anterior mediastinum.

In our study, the choice of surgical procedure depended on the training experiences of difference surgeons. The success of surgery might have been related to the surgeons. Nevertheless, logistic regression analysis showed that the surgical method (*p* = 0.426) and surgeon experience (*p* = 0.719) were not independent prognostic factors for these patients.

### Limitations

This was a retrospective study performed in a single centre. To confirm these findings, randomized controlled multicentre clinical trials are needed.

## Conclusions

The single-port VATS procedure was not inferior to the three-port VATS procedure. The single-port VATS procedure was a feasible choice for mediastinal cystectomy.

## Abbreviations

BMI: Body mass index,
FEV1.0: Preoperative symptom, forced expiratory volume in one second
FEV1.0/FVC: Forced expiratory volume in one second/forced vital capacity
VATS: Video-assisted thoracic surgery
